# Structure, Folding and Stability of Nucleoside Diphosphate Kinases

**DOI:** 10.3390/ijms21186779

**Published:** 2020-09-16

**Authors:** Florian Georgescauld, Yuyu Song, Alain Dautant

**Affiliations:** 1 Department of Chemistry and Chemical Biology, Northeastern University, Boston, MA 02115, USA; 2 Department of Neurology, Massachusetts General Hospital and Harvard Medical School, Charlestown, MA 02129, USA; ysong13@mgh.harvard.edu; 3 Institut de Biochimie et Génétique Cellulaires, Univ. Bordeaux, CNRS, IBGC, UMR 5095, F-33000 Bordeaux, France

**Keywords:** nucleoside diphosphate kinase structure, oligomeric state, quaternary structure, protein folding, protein stability, histidine kinase

## Abstract

Nucleoside diphosphate kinases (NDPK) are oligomeric proteins involved in the synthesis of nucleoside triphosphates. Their tridimensional structure has been solved by X-ray crystallography and shows that individual subunits present a conserved ferredoxin fold of about 140 residues in prokaryotes, archaea, eukaryotes and viruses. Monomers are functionally independent from each other inside NDPK complexes and the nucleoside kinase catalytic mechanism involves transient phosphorylation of the conserved catalytic histidine. To be active, monomers must assemble into conserved head to tail dimers, which further assemble into hexamers or tetramers. The interfaces between these oligomeric states are very different but, surprisingly, the assembly structure barely affects the catalytic efficiency of the enzyme. While it has been shown that assembly into hexamers induces full formation of the catalytic site and stabilizes the complex, it is unclear why assembly into tetramers is required for function. Several additional activities have been revealed for NDPK, especially in metastasis spreading, cytoskeleton dynamics, DNA binding and membrane remodeling. However, we still lack the high resolution structural data of NDPK in complex with different partners, which is necessary for deciphering the mechanism of these diverse functions. In this review we discuss advances in the structure, folding and stability of NDPKs.

## 1. Introduction

Nucleoside diphosphate kinases (NDPKs) are multifunctional oligomeric proteins [1] encoded in humans by the nucleotide metabolism enzyme (NME) genes, also called NM23 [2,3]. They are present in all three domains of life (bacteria, archaea and eukaryotes) and in some viruses. Most bacteria have only one cytosolic isoform, while eukaryotes can present up to 10 different isoforms [2,3], located in the cytosol, nucleus or mitochondria [1,4]. NDPKs are enzymes responsible for the transfer of the γ-phosphate between nucleoside triphosphates and nucleoside diphosphates via a ping-pong mechanism involving a phospho-histidine intermediate of high energy [5,6]. Inside cells, the donor of phosphate is mainly the ATP, the acceptor often being the GDP. NDPKs thus produce GTP for elongation in protein synthesis, signal transduction and microtubule polymerization; CTP for the synthesis of lipids; UTP for the synthesis of polysaccharides and NTPs or dNTPs for the synthesis of nucleic acids. In addition to these well-established metabolic functions [7,8,9,10], other important roles have been identified: some isoforms can bind DNA [11,12], act as metastasis suppressors [1,13,14], participate in membrane remodeling through interaction with dynamins [1,15] or influence cytoskeleton dynamics [16,17].

The involvement of NDPKs in different aspects of metastasis is particularly important. The interaction between NDPKs and cytoskeletal components such as actin, actin binding proteins, intermediate filaments and tubulin has been described by several groups and is thought to be highly relevant in the context of metastasis, partially due to the well-established role of cytoskeleton in cell motility and metastasis spread [16,17]. In addition, its ability to interact with other key players in cancer metastasis, such as small GTPases, G protein coupled receptors (GPCRs), ion channels, extra cellular matrix and vascular basal membrane proteins, suggests that NDPK is an important upstream regulator of multiple cancer metastasis signaling pathways [1,18]. Therefore, it is perhaps not surprising that NDPKs can also act as metastasis activators in certain cancer types [13,14,19]. In order to decipher the different roles of NDPKs and their underlying molecular mechanisms, detailed structural analyses of NDPKs need to be performed in various cellular systems in the presence or absence of specific interactors.

The first X-ray crystal structure of NDPK was solved for the hexamer enzyme from the amoeba *Dictyostelium discoideum* (*D. discoideum*) (1ndk) [20]. One year later, a second type of structural assembly was described (type I tetramer) with deposition of the structure from the bacterium *Myxococcus xanthus* (*M. xanthus*) (2nck) [21]. It took 14 more years before a third type of assembly (type II tetramer) was documented by solving the structure of NDPK from the bacterium *Escherichia coli* (*E. coli*) (2hur) [22]. Finally, a crystal structure for stable dimers was solved in 2012, constituting the fourth different type of quaternary structure for members of the NDPK family [23]. So far, 162 structures from 30 different species have been deposited within the Protein Data Bank (PDB) which fall into one of these four classes. Importantly, for all of them the monomers present a ferredoxin fold (also known as αβ sandwich), in the presence or absence of ligands. The vast majority of deposited structures are hexamers, twelve belong to tetramer type I, one belongs to tetramer type II and two are isolated dimers [23,24,25] (Table 1). The structure of most tetramers was recently solved by a structural genomics approach yet to be published (Table 1). Today, high resolution structures are available for each type of assembly: hexamer 1.25 Å (4c6a, *D. discoideum*) [26], tetramer type I 1.37 Å (3ztp, *Aquifex aeolicus*) [27], tetramer type II 1.62 Å (2hur, *E. coli*) [22] and dimer 2.30 Å (3vgs, *Halomonas* sp. 593) [23]. NDPK crystallizes in a wide range of crystallization conditions, with a pH range from 4.5 (1wkl/4kpc) to 9 (3ztq), in complex with nucleotides or analogs and dinucleotides. Ligands and buffer molecules are incorporated into the active site from the crystallization solution (Mg^2+^, SO_4_^2−^, PO_4_^3−^, citric acid) [28]. In NDPK crystal forms, the volume occupied by the solvent (calculated using the CCP4 program Matthews_coef [29]) varies from 31% in hexamers (1hiy) to 75% in tetramers (3vgs). Recently, the structure of a complex formed by NDPK bound to ciliary doublet microtubules was determined by cryo-electron microscopy (cryo-EM) (6u42) [30]. The availability of these different solved structures provides a wealth of information. In this review we will focus on the NDPK structure in the view of the monomer, and its different types of physiological assemblies: dimers, tetramers and hexamers. We will discuss how monomers acquire their native conformation during the folding phenomenon and how they assemble into active complexes. Finally, the contribution of quaternary structure to the stability of NDPK will be reviewed, as well as the contribution of structural biology toward understanding the different NDPK functions.

## 2. NDPK Monomer Structure

### 2.1. The α/β Domain

**Figure 1:**
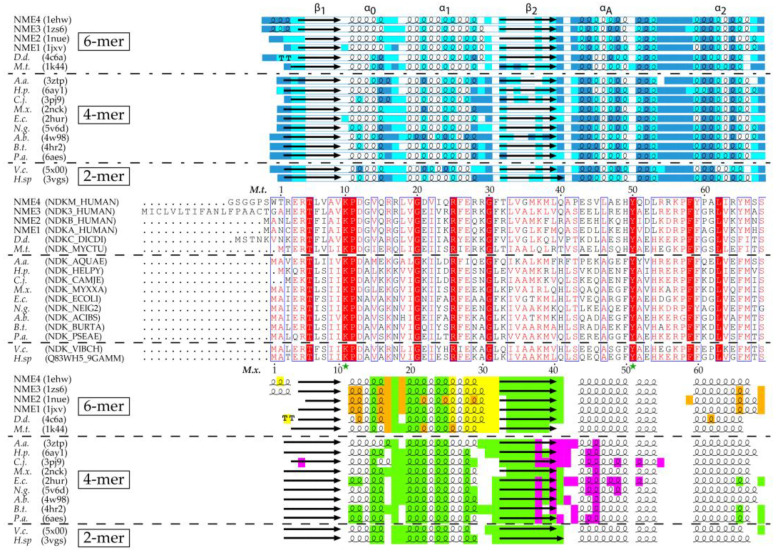

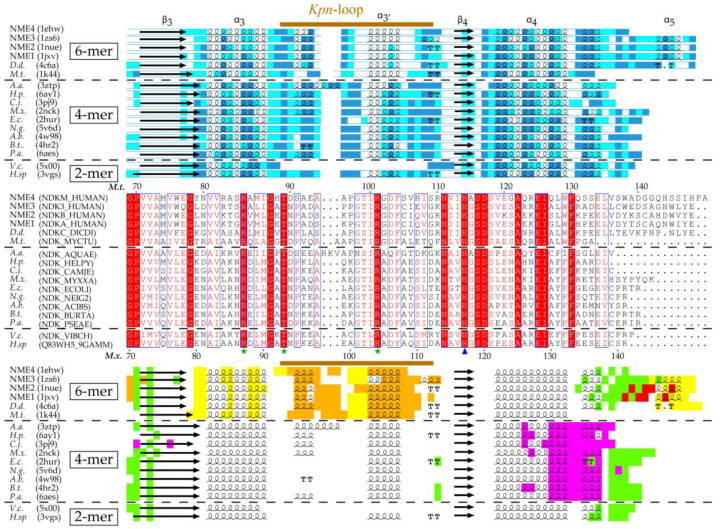
Secondary structure, residue accessibility, sequence and interface alignments of hexamer, tetramer and dimer NDPKs (nucleoside diphosphate kinases) with known 3D structure. (i) The relative accessibility and the secondary structure of each residue were calculated using DSSP (Database of Secondary Structure of Proteins) [31]. The accessibility is rendered as colored boxes (marine, cyan and white for accessible, intermediate and buried, respectively). α-helices, β-strands and strict β-turns are displayed as squiggles, arrows and TT letters, respectively. The conventional names of the NDPK secondary structure elements and the *Kpn*-loop are indicated above. (ii) On sequence alignment, strictly conserved residues are in white on a red background while similar residues are in red on a white background with blue frames (with numbered residues of *M.t.* and *M.x.* marked above and below the alignment, respectively). The active site residues and the catalytic histidine are indicated by a green star, and a blue arrow, respectively. (iii) The various interfaces are highlighted in different colors: green for the common-dimer interface, maroon and yellow for the trimer interfaces, red for the C_Term_ trimer interface, magenta for tetramer interface. The interfaces were identified using PISA (Proteins, Interfaces, Structures and Assemblies) [32]. The figure was drawn with ESPript3 [33]. (*NME 1 to 4*, Non Metastatic human isoforms 1 to 4, *D.d.*, *D. discoideum*; *M.t.*, *M. tuberculosis*; *A.a.*, *A. aeolicus; H.p.*, *H. pylori; C.j.*, *C. jejuni*; *M.x.*, *M. xanthus*; *E.c., E. coli*; *N.g.*, *N. gonorrhoeae; A.b.*, *A. baumannii; B.t.*, *B. thailandensis*; *P.a., P. aeruginosa*; *V.c.*, *V. cholerae*; *H.* sp, *Halomonas* sp. *593*). The PDB and Uniprot Ids are in parentheses.

For all NDPKs, the polypeptide chain forming the base subunit adopts a similar fold regardless of the organism considered or of the presence or absence of ligands. Each subunit is made up of approximately 140 amino acids (Figure 1), with variations for the 5 amino acids at the N-terminus and the 10 to 15 amino acids at the C-terminus.

**Figure 2:**
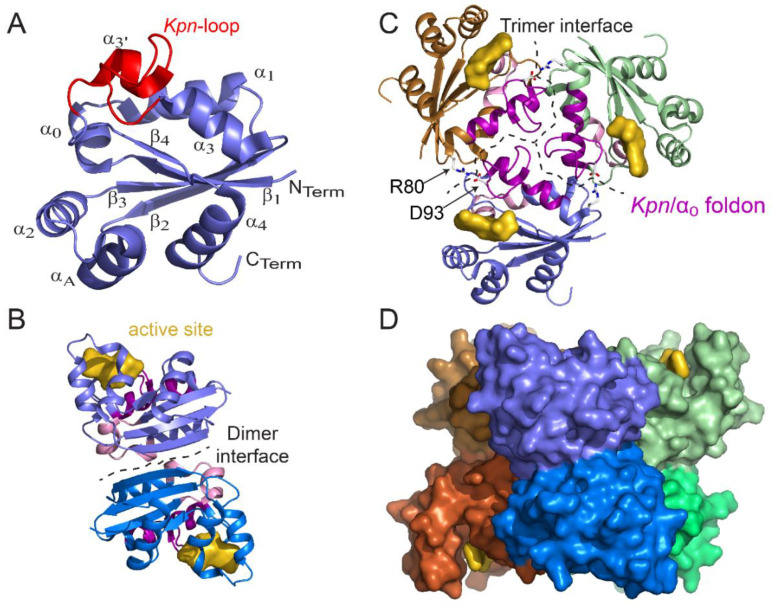
Structure of monomer, dimer, trimer, and hexamer of NDPK from *M. tuberculosis* (Figure adapted with permission from [36]). (**A**) View of the monomer with labeled secondary structural elements. (**B**) Side view of a dimer showing the dimer interface (residues 17, 19–21, 23, 24, 27, 33–38, and 72) and the active site pocket (K10, Y50, R104, N114, H117, S119, and E128). (**C**) Top view of a trimer. At the trimer interface (residues 16, 25, 28–31, 79, 80, 83, 84, 87, 88, 93–96, 98–102, and 105–110), the *Kpn*/α_0_ foldon (the *Kpn*-loop and the α_0_ helix are colored magenta and pink, respectively) and the R80–D93 salt bridge (sticks) are involved in hexamer assembly. The trimer stacks in a “head-to-head” manner and not in a “head-to-tail” manner such that the *Kpn*/α_0_ foldon is exposed on either side of the hexamer. (**D**) Side view of the surface of the six-color hexamer Mt-NDPK. The active site is colored yellow. In panels B and C, the chains are colored as in panel D. All structure figures were drawn using PyMOL molecular graphic system [38].

The core of the NDPK subunit forms an α/β domain of approximately 90 residues, comprising a central β-sheet of 4 antiparallel strands (β_2_β_3_β_1_β_4_ topology) surrounded on each side by α-helices (Figure 2A). The short α_0_ helix between the β_1_ strand and the α_1_ helix constitutes the bottom of the active site pocket. 

### 2.2. Structural Specificities of NDPK

**Figure 3:**
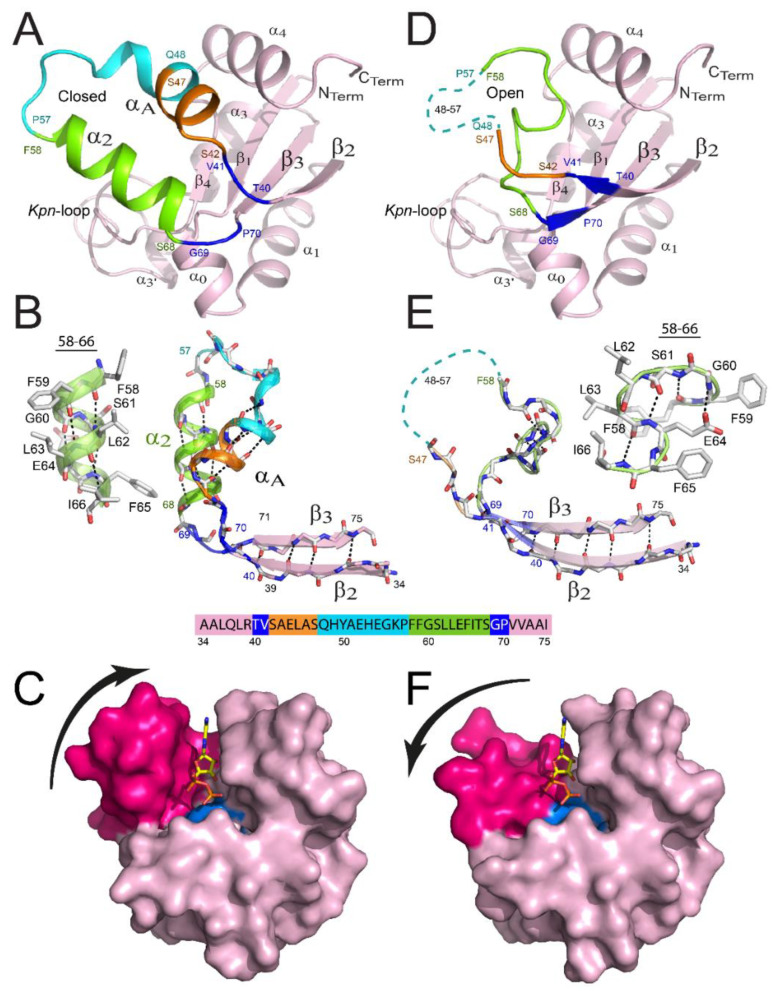
Remodeling of the binding site of NDPK from *M. tuberculosis.* View of a monomer presenting a closed structure (**A**) versus the remodeled one (**D**). The β_2_α_A_α_2_β_3_ region is remodeled in the latter monomer, and details are provided in panels (**B**,**E**), respectively. Residues 40–41, 42–47, 48–57, 58–68, and 69–70 are colored blue, orange, cyan, green, and blue, respectively. The region covering residues 48–57 is not visible in the X-ray structure for monomers and is shown as a cyan dashed line in panels D and E. The α_2_ helix (**B**) and the successive β-turn structures (**E**) are shown as cartoons and ball-and-stick structures. The surface views of the monomer are displayed in the closed (**C**) and open (**F**) conformations. α_A_ and α_2_ helices are colored magenta and the catalytic His117 is colored cyan. ADP is bound in the active site by homology with NDPK-A. (Figure adapted with permission from [41]).

The structure of the NDPK subunit has two additional characteristics: the “*Kpn*-loop” and the C-terminal residues extending after the α_4_ helix, which complete the fold [6]. The “*Kpn*-loop” is located between the α_3_ helix and the β_4_ strand (Figure 2A and Figure 3A). In *D. discoideum*, it is made up of residues 96–120 and comprises several types of helical structural elements with an α-helix (α_3′_ which extends the α_3_ helix and one turn of 3_10_-helix. The *Kpn*-loop takes its name from the “Killer of prune mutation” on the *awd* gene encoding a Drosophila NDPK [6]. On its own, the *Kpn* mutation does not cause a particular phenotype, but becomes lethal when it is associated with a dysfunction of the plum gene which codes for a protein exhibiting phosphodiesterase activity [34,35]. This mutation corresponds to the substitution by a serine of a proline conserved in all NDPKs: Pro100 for *D. discoideum*, Pro97 for Drosophila, and Pro96 for human NDPK-A (NME1). This loop is positioned at the active site of NDPKs and plays a role in surface contacts leading to protein oligomerization. The destabilizing effect of mutations inside the *Kpn*-loop will be discussed in the following section dedicated to the NDPK’s stability. The C-terminal part (that is, the residues extending after the α_4_ helix) is one of the most mobile and variable areas of NDPK and is involved in the dimerization and trimerization of the majority of hexamers. Its length varies from 18 residues in human isoforms to 4 residues in *Mycobacterium tuberculosis* (*M. tuberculosis*) [36,37]. In contrast, the C-terminus is shorter in tetramers (see alignment, Figure 1) and is not involved in the quaternary structure.

### 2.3. Active Site and Substrate Fixation

The active site comprises the nucleotide binding site plus the strictly conserved nucleophilic histidine 117 (numbered from the sequence of *M. tuberculosis*) (Figure 2A). Each monomer inside NDPK complexes presents one active site and functions independently. The catalytic histidine in the active site has been identified by direct protein sequencing, site mutagenesis and X-ray crystallography (reviewed by [5,6,39,40]).

Histidine 117 is located in the center of the strand β_4_, a very short strand which follows the *Kpn*-loop and connects it by a “turn” forcing residue 115 (Leu or Ile in most NDPK in eukaryotes) to adopt an unfavorable conformation in order to maintain the accessibility of the substrate to the active site. Indeed, if this residue adopted a β-strand conformation, its side chain would be in contact with that of His117, thus blocking its access to the substrate. The accessibility of His117 is thus preserved by the formation of a hydrogen bond between the NH of the main chain of Leu115 and the side chain of Asp12, which is conserved in the sequences of NDPKs. His117 is also constrained by other types of interactions in order to maintain its accessibility and ensure responsive orientation. The numerous structures of NDPK resolved in the presence of different substrates show that all nucleotides bind in the same way to the catalytic site. The helical hairpin α_A_-α_2_ and the *Kpn*-loop form a kind of clamp in which the nucleotide is fixed. The base of the nucleotide is oriented towards the surface of the molecule and stacks on the conserved phenylalanine 58, through formation of an interaction by aromatic “stacking”. The phosphate part is buried and oriented towards the catalytic histidine. The interactions between the protein and the phosphate group involve the side chains of the conserved residues Lys10, Tyr50, Arg86, Thr92 and Arg104 (Figure 1). Substitution of these residues decreases the enzyme activity [42]. Finally, the ribose-phosphate moiety adopts a “folded” conformation that allows the oxygen connecting the β-phosphate and the γ-phosphate to form a hydrogen bond with the 3′-OH of the sugar, which is 3′-OH itself involved in hydrogen bonds with Lys10 and Asn114. These bonds are essential for catalytic activity, the 3′-deoxynucleotides being poor substrates for NDPK [5,43]. The divalent cation, usually magnesium, binds to α- and β-phosphates and is necessary for the catalytic reaction. The structure of *M. xanthus* NDPK (1nlk) resolved in the presence of ADP without Mg^2+^ shows that cations are not necessary for substrate binding, the phosphate part being disordered in the structure [21]. In contrast, in complexes containing Mg^2+^, the cation forms six bonds with the oxygens of the phosphates or with water molecules, stabilizing and keeping the phosphate group in a reactive position [6,44].

Recently, an important remodeling has been revealed for one trimer inside the NDPK hexamer from *M. tuberculosis* (Figure 3D–F), the second trimer presenting the classical unmodified conformation (Figure 3A–C) [41]. The remodeling was identical for all three subunits of the trimer, covering residues 40–70 and contributing to: (i) the full disappearance of α_A_-α_2_ helix hairpin; (ii) the appearance of a new structure formed by successive β-turns of type I and II covering residues 58–66; (iii) the increase of lengths for strands β_2_ and β_3_; and (iv) the absence of stable structure for the region covering residues 48–57 that are no longer visible in the electron densities. As a result, the cleft of the binding site became more open, rendering the catalytic His117 significantly more accessible to substrates. Importantly, hydrogen deuterium exchange mass spectrometry (HDX-MS) revealed that residues 40–70 are the only ones inside the hexamer that are fully solvent accessible, indicating that an equilibrium between these two conformations is possible (Figure 3C,F). Such behavior was also noticed for the WT (wild type) as well as for two point-mutants (R80A; R80N) for which the mutation site is not part of the remodeled region of the protein [41]. It is tempting to speculate that such equilibrium occurs in vivo, explaining how bulky substrates (e.g., nucleic acids or peptides) access the catalytic His117.

Twenty-six crystal structures of viral NDPK (apo and in complex with various nucleotides) have been determined at resolutions ranging from 1.5 Å to 2.8 Å [45]. They are all hexamers and differ from eukaryotic NDPKs by presenting shorter *Kpn*-loops and two viral specific residues, near the active site. These differences structurally explain their stronger affinity for both deoxynucleotides and pyrimidine nucleotides, necessary for the replication of an AT-rich viral genome in a thymidine-limited host environment.

## 3. NDPK Quaternary Structure

### 3.1. Dimer: The Basic Subunit of NDPK Oligomer

All functional NDPKs so far described in the literature are oligomers [27,37,46]. Eukaryotic NDPKs are hexamers, while prokaryotic NDPKs are hexamers or tetramers [6,46]. Regardless of the oligomerization state of these enzymes, the oligomers are always constructed from a dimer formed by two subunits assembled head to tail [6,46]. The dimer interface is carried out through the combination of two β_2_ strands, one from each subunit, thus allowing the formation of a single antiparallel sheet of 8 β-strands (Figure 2B). Comparisons between dimers from the hexamer (of *D. discoideum*) or the type I tetramer (from the deltaproteobacteria *M. xanthus*)*,* and dimers from type II tetramer (from the gammaproteobacteria *E. coli*), show that the root mean square deviations (rmsd) for the structurally equivalent Cα carbons were 1.4 Å and 1.1 Å, respectively, clearly demonstrating that all NDPK oligomers share the dimer as basic structural unit. Such a NDPK dimer was found in *Halomonas* sp. *593* (a halophilic bacterium) using size-exclusion chromatography coupled with multi-angle laser light scattering (SEC-MALLS) [47] and the resolution of the structure confirmed its dimeric state (3vgt and 3vgs) [23]. Interestingly, the WT NDPK was able to form a type I tetramer through a single mutation E134A, situated at the tetrameric interface (3vgu and 3vgv) [23]. Recently, the second structure of a WT dimer was solved for *Vibrio cholerae* NDPK (5x00) (Table 1). Although the α_3′_ helix and the *Kpn*-loop were unobserved, the best superimposition with other NDPKs resulted in a rmsd of 0.41 Å for the monomer and 0.54 Å for the dimer of *E. coli* NDPK.

### 3.2. Hexamer and Tetramer of NDPK

Functional NDPK oligomers are formed from the assembly of dimers [6,46]. The dimers self-assemble, two or three times into tetramers or hexamers [22]. One main difference between hexameric and tetrameric NDPKs comes from the C-terminal segment (Figure 1), which is longer for most hexameric NDPKs: it brings the two dimer subunits into contact and also participates in their trimerization. A C-terminal segment of 15 residues on average is responsible for an additional burial of the protein surface of about 30% (or about 300 Å^2^) compared to the enzymes which lack such segments and which strongly stabilizes the hexamer [37,48]. In the NDPK from *D. discoideum* the P100S mutation combined with the successive deletion of the last 5 C-terminal amino acids leads to a loss of activity as well as the dissociation of the enzyme into dimers [48,49]. That being said, the shorter C-terminus is not sufficient to explain this difference in oligomerization and exceptions exist: the structures from *M. tuberculosis* [50] and from *Bacillus halodenitrificans* [51] are hexameric although their sequence contains a short C-terminal segment. For these short NDPKs other mechanisms exist to stabilize the hexamers as revealed by the structural analysis of destabilized mutants (see chapter on NDPK stability). For eukaryotes containing different NDPK isoforms, it has been recently reported that their hexamers are heterohexamers (containing at least two different isoforms); isoform-pure hexamers (also known as homohexamers) scarcely exist in vivo [52].

In the tetrameric structures of types I and II, the C-terminus is shorter and only interacts with the neighboring subunit inside the same dimer [46]. By assembling into hexamers or tetramers, dimers interact through different interfaces and details concerning specific residues involved in different interfaces were discussed by Moynié and colleagues [22]. In solution, the NDPK tetramers from *E. coli* dissociate into dimers when the concentration of the enzyme decreases [53], raising the question of the existence of an intracellular equilibrium between dimers and tetramers. It would be interesting to understand how the shift from tetramer to dimer affects the enzymatic activity, though both oligomers present strictly identical catalytic sites.

The type I tetrameric interface of the NDPK from *M. xanthus* is characterized by a small buried surface area (bsa: 500 Å^2^, 6.6% of the accessible surface area (asa) of the monomer). Surface areas have been calculated using PISA [32]. Such an interface was not present in the *E. coli* NDPK crystal contacts, whereas the opposite face of the dimer was buried (bsa: 470 Å^2^, 6.2% of the asa of the monomer), leading to the proposal of a type II tetramer. In the case of small biological interfaces, it is very difficult to distinguish the contacts which are biologically relevant from the lattice contacts. As a result, the software dedicated in crystallography to identify interfaces could fail in deciphering the real assembly. As part of structural genomic programs, several tetramer structures were recently deposited at the PDB (Table 1). These structures are type I tetramers and displayed a modest bsa about 365–490 Å^2^. Interestingly, one of them (3pj9) displays dimeric and tetrameric interfaces with similar bsa 680 Å^2^ and 610 Å^2^, respectively, and forms a type I like tetramer. In light of all deposited X-ray crystal structures on NDPK tetramers, determining the real biological interfaces based only on the crystal structure seems risky, and thus complementary approaches such as cross linking mass spectrometry or NMR are needed to reliably identify these interfaces.

### 3.3. The NME Family

Ten human genes (NME1–10) encode isoforms of NME [2,3] where the gene products differ in their functions and subcellular localizations (for a review see [54]). Structures are available for NME1 (1jxv, 1ucn, 2hvd, 2hve, 3l7u, 4eno, and 5ui4), NME2 which is also a transcription factor (1nsk, 1nue, 3bbb, 3bbc, and 3bbf), NME3 involved in DNA repair (1zs6), the mitochondrial NME4 (1ehw) that interacts with cardiolipin-containing membranes [55], and FAP67 (an analog to NME7) (6ua2).

## 4. NDPK Folding

Mutants of NDPKs have provided insights into the dynamics of folding and functional diversity. For example, in addition to the well-characterized nucleoside kinase activity, the human isoform A (further called NDPK-A or Nm23-H1) is the first metastasis suppressor gene identified [56]. Although mutations are rare for this isoform, an S120G point mutation was found in several advanced neuroblastomas but not in limited-stage tumors [57], and since then, intensive research has been performed on NDPK-A (see recent review by [13]). First studies of the mutated protein showed an altered interaction with cellular partners in vivo, and its biochemical characterization showed that conversely to the WT which only form hexamers, the mutant formed hexamers as well as sub-hexameric species as monomers, dimers or tetramers [58]. In this context, the hypothetical aberrant folding and assembly of NDPK-A was studied by several groups [59,60,61,62].

Studies of recombinant S120G NDPK-A showed reduced thermal stability to denaturation, when compared to the wild type protein [61]. More interestingly, while the WT protein denatured in urea, renatured and associated into active hexamers by dilution in vitro, the S120G mutant remained as monomeric, as a molten globule folding intermediate [61]. It was also noticed that the folding defect of the S120G mutant was corrected in vitro by the presence of ATP (Ioan Lascu, personal communication during the 3rd international conference on NDPK, Bordeaux 1999) and two mechanisms through which the folding defect was corrected were discovered a few years later. The first mechanism involves phosphorylation on its catalytic histidine, which completely corrected the folding defect. It was shown that after phosphorylation by ATP or phosphoramidate, the S120G mutant renatured and assembled rapidly in high yield [62]. Binding of nucleotides (ADP, AMP-PNP) had no effect on folding, but increased the stability of native hexamers. It was proposed that protein phosphorylation was the physiological mechanism that explains why the S120G mutant is active in neuroblastomas and why it is produced as native hexamers in *E. coli*, despite the folding defect observed in vitro. The second mechanism involves the chemical chaperone trimethylamine-N-oxide (TMAO), which was able to correct the folding defect of the S120G mutant through its unfavorable interaction with the main chain of the protein [59]. This complements the first mechanism based on phosphorylation, as TMAO acts on both folding and assembly of monomers. The native hexameric structure of the S120G mutant did not show any difference from the WT, both for the overall structure and in the vicinity of the mutation [60]. The strong structural similarities between the two agreed with the comparable kinase activities [61] and indicated that the specificities of the S120G mutant cannot be attributed to NDPK activity. This suggests that other properties of this multifunctional enzyme may be affected by the mutation, such as the interaction with its substrates: proteins and/or DNA. Finally, it was shown in vitro that S120G hexamers lost their native conformations and formed beta amyloids [63] which might play a role in cancer. WT NDPK-A also formed amyloid aggregates when incubated at temperatures above 60 °C. However, NDPKs from other organisms were not able to form such aggregates, suggesting that the molten-globule-type folding intermediates, which are unique for human NDPK-A, may be required in the formation of amyloid structures. The presence of such amyloid structures in cancer biology has been proposed [64], suggesting that cellular factors may exist to produce molten globule intermediates in addition to high temperature incubation.

The folding and assembly process of the NDPK from *D. discoideum* was also studied [48,65,66] by classical biochemistry experiments to show that dimeric mutants of NDPK were unable to autophosphorylate, suggesting that dimers may not be able to bind the substrate. We recently confirmed this hypothesis using HDX-MS technology at the peptide level. Characterizing stable dimers from *M. tuberculosis*, we showed that they were native-like, except for a part of the binding site we called *Kpn*/α_0_ subdomain which remained unfolded [36]. These dimers were unable to get phosphorylated presumably due to the fact that their binding sites are not fully constituted. Only after assembly into hexamers do the *Kpn*/α_0_ subdomains get structured and the NDPK becomes enzymatically functional. That constitutes a scenario different from the one observed for tetramers, in which the *Kpn*/α_0_ subdomain is folded in both dimers and tetramers. Finally, several studies used NDPK as model for understanding the effect of osmolytes on monomer folding or their assembly [59,67,68] and demonstrated that folding of halophilic NDPKs required high salt concentration [67].

## 5. NDPK Stability

Studies on the differential stability for NDPKs with mutations that do not affect basal enzymatic activity and natural variants have been valuable for elucidating mechanisms for stabilization of complex structures. Initial studies were performed on the stability of NDPKs because of the discovery of defective mutants involved in tumor metastasis in cancer patients [57,58] and in the aberrant larval development in *Drosophila* [69]. Interestingly, these mutations destabilized the structures of the monomers (e.g., the human S120G HA isoform) [59,61,62] or the hexamers’ quaternary structure (e.g., the *Drosophila* P97S mutant) [69] without significantly affecting the enzymatic activity. Pioneering microcalorimetry studies used NDPK as a model oligomeric protein to understand the effect of quaternary structure on the complex stability [70]. More recently, additional studies used NDPK complexes as model proteins to understand how the stability of homo-tetramers or homo-hexamers is improved for extremophiles like halophiles [71,72,73,74], thermophiles [27,75] and psychrophiles [76]. Often, they used the same strategy to show through mutational analysis how different residues affect the overall stability of NDPK complexes. Other studies on NDPK stability concerned proteins from trypanosomatid parasites of subfamily *Leishmania*, which are secreted and must be stable enough for performing their function in potentially hostile conditions [77,78,79,80]. All these different studies stressed out the key role of the specific structures of NDPK (the *Kpn* loop and the C-terminal segment) in the stabilization of the quaternary structure. Finally, because NDPKs are an old family of proteins, they also provide an opportunity for studying the stability of proteins during evolution [67,81,82]. These studies concluded that ancient sequences of NDPK were thermally extremely stable and needed a restricted alphabet of only 13 different amino acids to be enzymatically active [83,84].

**Figure 4:**
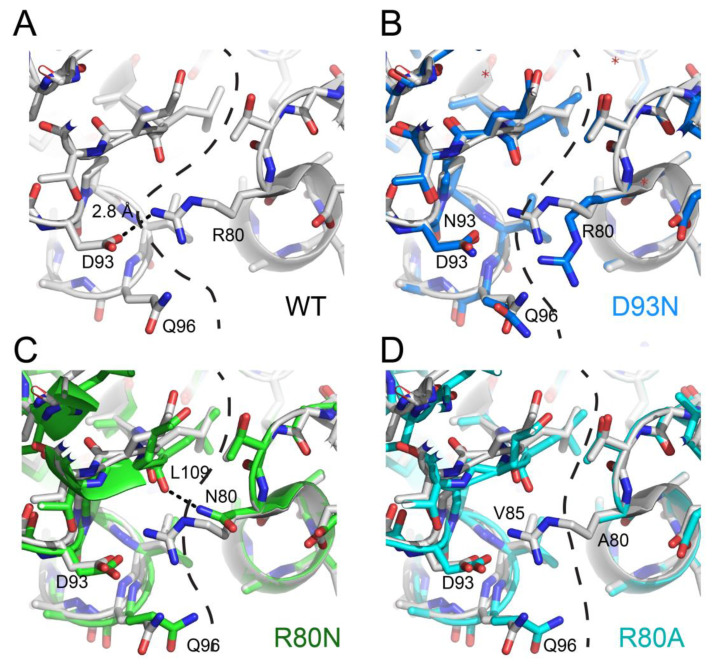
Stabilizing mechanisms induced by mutations at subunit interfaces of *M. tuberculosis* NDPK. (**A**) WT (wild type; white), (**B**) D93N (marine) vs. WT (white), (**C**) R80N (green) vs. WT (white), and (**D**) R80A (cyan) vs. WT (white).

Because of the limited space, we will focus only on recent articles using available structural data to explain some mechanisms through which the NDPK complexes are stabilized. *Mycobacterium tuberculosis*, the pathogen responsible for tuberculosis, escapes the immune system by blocking inside macrophages the maturation of phagosomes supposed to destroy it [85,86]. This complex mechanism requires high stability of bacillus proteins, including that of NDPK [87]. As mentioned in the section describing the NDPK structure, the majority of NDPKs present at the C-terminal a segment of 7 to 16 amino acids which interacts with two neighboring subunits and these interactions participate in the overall stability of the hexamers. In *M. tuberculosis*, NDPK does not have this segment, but their hexamers are thermostable and present a T_m_ of 76 °C [50]. Combining structural biology and point mutants, we showed that their stabilization is due neither to the existence of additional hydrophobic patches, nor to hyper-stabilization of the monomers [37], but occurred through a strengthening of the ionic interactions between neighboring subunits (Figure 4A), which compensated for the absence of the stabilizing segment at the C-terminal. The stabilizing role of the six salt bridges (D93-R80) was shown by the study of the mutant D93N which loses these bonds and exhibits a 28 °C destabilization, with a T_m_ of 48 °C (Figure 4B). Unexpectedly, the R80N mutant (for which the six D93-R80 salt bridges are destroyed) exhibited a stability close to that of the wild-type protein, with a Tm of 69 °C. The 1.9 Å resolution of the R80N mutant structure provided the mechanism: the side chain of N80 forms two new strong hydrogen bonds with two neighboring subunits (Figure 4C) [28]. Another stabilizing mechanism was discovered by analyzing the structure of R80A mutant, which also shows little destabilization (T_m_ of 69 °C), despite the disappearance of ionic bonds [41]. In this case, stabilization was generated through the formation of a hydrophobic patch including the hydrophobic Ala93 (Figure 4D). Studies on tetrameric NDPK from the hyperthermophile *Aquifex aeolicus* showed the existence of still another stabilizing mechanism: the formation of disulfide bridges between the subunits [27]. Finally, Pédelacq and collaborators showed that hyper-stabilization for archaeal NDPK from *Pyrobaculum aerophilum* is realized through appearance of additional subdomains [75]. Together, these studies underscore the critical role of quaternary structure in the stability of NDPK and showed that during evolution, different stabilization mechanisms can develop inside the same family of proteins.

The NDPK stability has also been studied in the presence of the chaotropic agents urea and guanidinium chloride (for a review see [46]). For these studies, the folded and the unfolded NDPK was incubated for 12 to 24 h in the presence of different amounts of denaturant to induce, respectively, the unfolding and the refolding of NDPK and enzyme activity or intrinsic fluorescence followed. Many of these studies compared the stability of the WT versus different mutants and concluded that: (1) most mutations had a destabilizing effect [49,59,61,62,69] and (2) the quaternary structure of thermophilic or hyper-thermophilic NDPK is stabilized by interactions between monomer subunits [36,41,46]. Quantifying thermodynamically the stabilization or the destabilization effect was not possible in most of these studies since the NDPK was not reaching equilibrium during unfolding/refolding but experienced a hysteresis phenomenon [66] and no thermodynamic parameter (ΔG, ΔH or ΔS) could be extracted. Interestingly, equilibrium was obtained for sub-hexameric species (monomers or dimers) and an attempt of their thermodynamic characterization was realized [37].

## 6. Concluding Remarks and Perspectives

With increasing knowledge of NDPK structures in various systems, with and without specific substrates, we have begun to understand the multifunctional roles of this unique family of proteins and their underlying molecular mechanisms. While the metabolic function of the “diphosphate kinase” component, exhibited by most isoforms, is now functionally and structurally well understood, the mechanistical understanding of NDPK in other important cellular functions such as DNA binding, protein phosphorylation, metastasis suppression, membrane remodeling and cytoskeleton dynamics is still limited. Such functions might require different states of oligomerization and dynamic structures in the presence of distinct interactors, which may explain cell-type and cell-stage specific functions of NDPK. For example, NDPK was suggested to interact with tubulin, one of the most abundant GTPases in the cell, and regulate microtubule dynamics, thus playing a general role in cell growth and differentiation. However, due to the lack of structural information concerning the interaction between NDPK and tubulin, the existence of a NDPK–tubulin complex has been controversial in the field up to very recently [88,89,90,91], when the cryo-EM structure of such a complex revealed further details about these interactions [30]. This study showed that a *Chlamydomonas* NDPK, also known as FAP67, bound twice within the 48 nm repeats on the seam of A-tubule [30]. FAP67, a protein of 380 residues, is an analog to NME7. The complex is formed by an N-terminal domain DM10 (residues 1–91) and two NDPK monomers covalently linked by an extended loop (residues 227–236). Interestingly, the two FAP67 molecules existed in two distinct conformations regarding the DM10 domain, perhaps due to the differences in neighboring Microtubule Inner Proteins (MIPs) and/or specific post-translational modification (e.g., acetylation on tubulin-K40). The authors speculated that the binding of NDPK in this case could ensure a high local GTP-tubulin concentration critical for axonemal extension in developing cilium and microtubule self-repair in mature cilium [30]. However, additional functions cannot be excluded, and further studies are required to understand why two copies of NDPK within the 48 nm repeat differ in their structure and whether this structural difference results in functional diversity. Similarly, we believe that structural analyses of different NDPK-substrate complexes in various cell types with complex protein/DNA profiles and unique distribution patterns (e.g., neurons), will help us understand the molecular mechanisms of other cellular functions of NDPK, as we decipher its transient or stable interactions with various partners involved in cancer metastasis, mitochondria function, synaptic remodeling, the nucleotide channeling and more. Because of incredible recent progress in structural biology methods, we believe structures with heterocomplexes containing different NDPK isoforms and complexes of NDPK/DNA and NDPK/lipids are within rapid reach.

## Figures and Tables

**Table 1:** Dimer and tetramer NDPK structures recently deposited in the Protein Data Bank.

Id	Species (Resolution, Å)	4D	Authors or References
**Betaproteobacteria**			
4dut	*Burkholderia thailandensis* (2.50 Å)	4-mer	[24]
4ek2	*Burkholderia thailandensis* (2.00 Å)	4-mer	[24]
4hr2	*Burkholderia thailandensis* bound to ADP (1.95 Å)	4-mer	Clifton, M.C.; Abendroth, J.A.
5v6d	*Neisseria gonorrhoeae* in complex with citrate (1.85 Å)	4-mer	Abendroth, J.; Mayclin, S.J.; Lorimer, D.D.; Edwards, T.E.
**Gammaproteobacteria**			
4s0m	*Acinetobacter baumannii* (1.92 Å)	4-mer	Sikarwar, J.; Shukla, P.K.; Kaur, P.; Sharma, S.; Singh, T.P.
4wbf	*Acinetobacter baumannii* (2.64 Å)	4-mer	[25]
4w98	*Acinetobacter baumannii* (1.43 Å)	4-mer	[25]
5yih	*Acinetobacter baumannii* (1.98 Å)	4-mer	Bairagya, H.R.; Sikarwar, J.; Iqbal, N.; Singh, P.K.; Kaur, P.; Sharma, S.; Singh, T.P.
5yol	*Acinetobacter baumannii* (2.2 Å)	4-mer	Singh, P.K.; Sikarwar, J.; Kaur, P.; Sharma, S.; Singh, T.P.
3vgs 3vgt	*Halomonas* sp. *593* WT (2.30 Å and 2.70 Å)	2-mer	[23]
3vgu 3vgv	*Halomonas* sp. *593* E134A mutant (2.30 Å and 2.50 Å)	4-mer	[23]
6aes	*Pseudomonas aeruginosa* (3.55 Å)	4-mer	Sikarwar, J.; Singh, P.K.; Sharma, S.; Singh, T.P.
5x00	*Vibrio cholerae* (3.06 Å)	2-mer	Agnihotri, P., Mishra, A.K.; Pratap, J.V.
**Epsilonproteobacteria**			
3pj9	*Campylobacter jejuni* (2.10 Å)	4-mer	Filippova, E.V.; Wawrzak, Z.; Onopriyenko, O.; Edwards, A.; Savchenko, A.; Anderson, W.F.
6ay1	*Helicobacter pylori* (2.05 Å)	4-mer	Edwards, T.E.; Dranow, D.M.; Lorimer, D.D.

## Abbreviations

asa	accessible surface area
bsa	buried surface area
cryo-EM	cryo-electron microscopy
DSSP	Database of Secondary Structure of Proteins
FAP67	Flagellar associated protein 67
HDX-MS	Hydrogen-Deuterium eXchange—Mass Spectrometry
*Kpn*	Killer of prune
NDPK	Nucleoside diphosphate kinase
NDPK-A	Nucleoside diphosphate kinase isoform A
NME	Nucleotide metabolism enzyme
PISA	Proteins, Interfaces, Structures and Assemblies
rmsd	root mean square deviation
SEC-MALLS	Multi-angle scattering of laser light coupled with size exclusion chromatography
TMAO	Trimethylamine-N-oxide
WT	Wild type
